# Ubiquitin choreography in nasopharyngeal carcinoma: USP18 scaffolds radioresistance

**DOI:** 10.1038/s41418-025-01616-2

**Published:** 2025-11-11

**Authors:** Francesco Napoletano, Rebecca Bertolio, Giannino Del Sal

**Affiliations:** 1 Department of Life Sciences, University of Trieste, 34127 Trieste, Italy; 2 International Centre for Genetic Engineering and Biotechnology (ICGEB), Area Science Park-Padriciano, 34149 Trieste, Italy; 3 IFOM ETS, the AIRC Institute of Molecular Oncology, Milan, Italy

**Keywords:** Cancer, Oncogenes

**Fig. 1:**
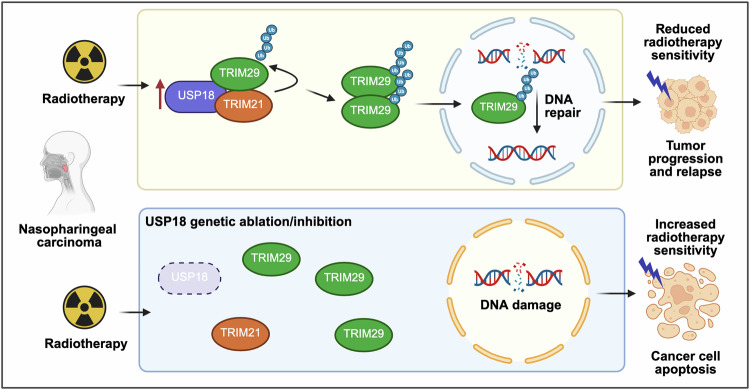
Role of the USP18–TRIM21–TRIM29 axis in nasopharyngeal carcinoma radioresistance. Following radiotherapy, USP18 is upregulated and scaffolds the formation of a protein complex in which TRIM21 ubiquitinates TRIM29. This modification promotes TRIM29 oligomerization and nuclear translocation, thereby enhancing the DDR, ultimately contributing to radioresistance and tumor progression. Genetic ablation of USP18 impairs TRIM21-TRIM29 interaction, and thus TRIM29-dependent DDR, sensitizing cancer cells to radio-induced DNA damage and consequent apoptosis. The image was made in BioRender.com.

Radiotherapy is a therapeutic mainstay for nasopharyngeal carcinoma (NPC), exploiting ionizing radiation to induce lethal DNA damage in cancer cells. Most patients respond well, yet approximately 20% relapse after curative treatment [1]. Accumulating evidence implicates activation of DNA damage repair (DDR) pathways in radioresistance [2, 3], however, the underlying mechanisms remain elusive. In the present issue of Cell Death & Differentiation, the manuscript entitled “USP18 promotes nasopharyngeal carcinoma radioresistance via TRIM29 oligomerization and ubiquitination” by Lin and collaborators uncovers that the Ubiquitin specific peptidase 18 (USP18) plays a key role in NPC radioresistance [4]. Mechanistically, USP18 acts as a scaffold protein, independently of its deubiquitinase (DUB) activity, promoting DDR that shields NPC cells from radiation-induced death (Fig. 1).

USP18 canonically targets proteins conjugated to the Ubiquitin-like protein interferon-stimulated gene 15 (ISG15), a post-translational modification (PTM) known as ISGylation, involved in anti-viral type I interferon (IFN-I) response [5]. By catalyzing the de-ISGylation, USP18 attenuates IFN-I signaling, for instance by de-ISGylating IFN-alpha 2 receptor (IFNAR2) with consequent inhibition of Janus kinase 1 (JAK1)/Signal Transducer and Activator of Transcription (STAT)-dependent IFN response. Beyond its established role in antiviral response, emerging evidence suggests that USP18 is also involved in tumour biology, although its contribution to cancer therapy response remains unclear. Through proteomic profiling and analysis of available transcriptomic profiles from NPC biopsies, Li et al. observed that tumors from patients who relapsed after radiotherapy exhibited high levels of USP18 expression, compared with those from non-relapsing patients [4]. Elevated USP18 levels also correlated with reduced radiosensitivity in NPC cells lines and poor clinical outcome in patients, suggesting that USP18 could serve as a bona fide biomarker of radioresponse. Functionally, CRISPR-mediated knockout of USP18 in irradiated NPC cell lines markedly increased unrepaired DNA damage, as shown by γH2AX foci and COMET assay, and apoptosis, and these phenotypes persisted even when IFN-I signaling was blocked pharmacologically. In mouse NPC xenografts, USP18 loss curtailed tumor growth upon irradiation (Fig. 1). Conversely, overexpression of USP18 suppressed radio-induced DNA damage and apoptosis, and enhanced tumor growth in mice. Through this elegant series of experiments, the authors established that USP18 drives NPC radioresistance operating outside its canonical immune regulatory function. Mechanistically, USP18 bound the tripartite motif containing 29 (TRIM29), a known activator of DDR, but unexpectedly did not promote TRIM29 de-ubiquitination. Instead, TRIM29 ubiquitination was increased, also by a catalytically inactive USP18 mutant, suggesting a possible structural, rather than enzymatic, function of USP18. Indeed, the authors found that USP18 serves as a scaffold for the E3 Ubiquitin ligase TRIM21, which in turn catalyzes K27-linked ubiquitination of TRIM29 at Lysine-561 (K561).

The type of Ubiquitin chain linkage dictates the outcome of Ubiquitin signaling [6]. K48-linked chains mark proteins for degradation, while K63 chains propagate signaling. The role of K27 chains is less understood, and this modification has been recently implicated in the regulation of immunity and DNA repair [7]. Lin and colleagues demonstrate that K27-linked ubiquitination of TRIM29 acts as a structural cue promoting its oligomerization and consequent nuclear translocation [4]. This modification stabilizes TRIM29 dimers through newly formed helical interfaces, as supported by molecular dynamics simulations. Once in the nucleus, oligomeric TRIM29 recruits DDR machinery to damaged chromatin, accelerating repair and attenuating radiation cytotoxicity (Fig. 1). Mutating TRIM29 into Arginine (K561R) disrupted ubiquitination, oligomerization, and nuclear import, thereby reversing the radioresistant phenotype (Fig. 1). Interestingly, K63-linked ubiquitination has also been implicated in the regulation of protein aggregates. For instance, a recent study in Drosophila models provided evidence that the HECT, C2 and WW Domain Containing E3 Ubiquitin Protein Ligases 1/2 (HECW1/2) control the function of the Fragile X Mental Retardation Protein (FMRP), by regulating its protein-protein interactions via K63-linked ubiquitination [8]. Taken together, this evidence supports a key role of non-degradative ubiquitination in the regulation of protein complex dynamics and function.

Importantly, Lin et al. also showed that, in vivo, the growth of NPC subcutaneous tumor xenografts promoted by USP18 overexpression upon irradiation was suppressed by TRIM29 knockdown, providing compelling evidence that the USP18–TRIM21–TRIM29 axis drives NPC radioresistance in preclinical models [4]. The TRIM protein family comprises over 70 E3 ligases characterized by RING, B-box, and coiled-coil domains [9]. TRIM29, however, lacks a canonical RING domain and has remained functionally enigmatic. The work by Lin and colleagues uncovers how TRIM29 can act as a DDR effector not through enzymatic activity but through conformational assembly [4]. Its coiled-coil domain enables higher-order oligomerization - an organizational principle shared with TRIM19/PML nuclear bodies [10] - while K27-linked ubiquitination further stabilizes these complexes. The result is a multimeric scaffold that recruits DDR mediators such as DNA-PKcs and BRCA1-associated factors, enhancing repair efficiency [11]. This mechanistic insight is consistent with prior observations that TRIM29 overexpression confers radioresistance in pancreatic and breast cancers, while its nuclear accumulation correlates with poor prognosis [12]. The USP18–TRIM21–TRIM29 cascade now provides a molecular rationale for these clinical correlations.

Catalytically inactive DUBs have previously been shown to act as scaffolds. For instance, USP18 was shown to stabilize the Stimulator of interferon genes (STING) protein via USP20-dependent deubiquitination, and to recruit the Mindbomb E3 ubiquitin protein ligase 2 (MIB2) to ubiquitinate and degrade the Gasdermin D protein GSDMD45 [13]. Nonetheless, the role of DUBs as scaffold regulators of the DDR and cancer resistance to therapy had not been explored. The evidence that USP18 can promote ubiquitination challenges the conventional view of DUBs, suggesting a more complex role in the coordination of E3 ligases, and Lin et al. now position USP18 as a central organizer of DDR signaling [4]. This finding also enriches the emerging concept of “ubiquitin code” of the DDR. K63- and K27-linked chains on histone H2A and other chromatin targets recruit key repair mediators such as 53BP1 [14]. Hence, by showing that TRIM29 undergoes a similar modification to facilitate repair complex assembly [4], the study by Lin et al. broadens the repertoire of ubiquitin-dependent DDR scaffolds.

From a therapeutic perspective, the findings have multiple implications. USP18 expression could serve as a predictive biomarker for radiotherapy response in NPC, identifying patients who might benefit from DDR inhibitors or radiosensitizers. Also, disrupting the USP18–TRIM21 interface could specifically dismantle the pro-repair scaffold without affecting USP18’s interferon-regulatory role, minimizing off-target immunotoxicity.

A key role in cancer progression and resistance to therapy is played by oncogenic signaling in response to microenvironmental biophysical cues, rewiring cancer cell metabolism and contributing to cancer cell adaptive response, including DDR, and immune evasion [15]. Emerging evidence underscores the role of microenvironmental stimuli also in NPC radioresistance [16], and in the regulation of ubiquitination signaling [17]. Within this complex tumor ecosystem, the findings by Lin et al. may thus represent an actionable link between microenvironmental cues and DDR-dependent resistance, an intriguing possibility that warrants further investigation. In addition, given the widespread expression of USP18 in other solid tumors, it will be important to explore whether the function of the USP18–TRIM21–TRIM29 axis exerts similar functions beyond NPC.
